# Tardus parvus waveforms in Doppler ultrasonography for hepatic artery stenosis after liver transplantation: can a new cut-off value guide the next step?

**DOI:** 10.1007/s00261-017-1358-2

**Published:** 2017-10-23

**Authors:** Bo-wen Zheng, Ying-yi Tan, Bin-sheng Fu, Ge Tong, Tao Wu, Li-li Wu, Xiao-chun Meng, Rong-qin Zheng, Shu-hong Yi, Jie Ren

**Affiliations:** 1 Guangdong Province Key Laboratory of Hepatology Research, Department of Medical Ultrasonics, The Third Affiliated Hospital of Sun Yat-sen University, 600 Tianhe Road, Guangzhou, Guangdong People’s Republic of China; 2 Guangdong Province Key Laboratory of Hepatology Research, Department of Liver Transplantation, The Third Affiliated Hospital of Sun Yat-sen University, 600 Tianhe Road, Guangzhou, Guangdong People’s Republic of China; 3 Department of Radiology, The Sixth Affiliated Hospital of Sun Yat-sen University, 26 Yuancun Erheng Road, Guangzhou, Guangdong People’s Republic of China

**Keywords:** Liver transplantation, Hepatic artery stenosis, Doppler ultrasonography

## Abstract

**Purpose:**

Considering the high false-positive diagnosis of the tardus parvus waveform (TPW) in Doppler ultrasonography (DUS) for hepatic artery stenosis (HAS) after liver transplantation (LT), this study aimed to determine clinical features and new cut-off values to help guide treatment.

**Materials and methods:**

This retrospective study was approved by an Institutional Review Board. A total of 171 LT recipients were included and underwent DUS and either computed tomography angiography or digital subtraction angiography with an interval < 4 weeks at least 1 month post-LT. The DUS of 69 patients exhibited TPW [defined as resistive index (RI) < 0.5 and systolic acceleration time (SAT) > 0.08 s]. A multilevel likelihood ratio (LR) analysis was used to explore new cut-off values for DUS. In addition, abnormal liver function was considered additional evidence (defined as any liver enzyme > 3-fold of the upper limit of normal level or 2-fold increased). The results were stratified into three categories, category 1 (subjects with traditional TPW), category 2 (subjects with traditional TPW and abnormal liver function), and category 3 (subjects with traditional TPW and abnormal liver function, or with new cut-off values), and the diagnostic performance of each category was analyzed.

**Results:**

The LR analysis revealed new cut-off values of RI < 0.4 (LR = 10.58) or SAT > 0.12 s (LR = 16.46). The false-positive rates for categories 2 and 3 were significantly lower (7.6% vs. 18.1%, *P* = 0.038; 1.9% vs. 18.1%, *P* < 0.001, respectively) than those for category 1, while the sensitivity for category 2 was significantly lower (41.8% vs. 74.6%, *P* < 0.001; 41.8% vs. 61.2%, *P* = 0.038, respectively) than that for categories 1 and 3.

**Conclusion:**

Using either (1) RI < 0.4 or SAT > 0.12 s, or (2) traditional TPW (RI < 0.5 and SAT > 0.08 s) in the presence of abnormal liver functions as the DUS criteria for HAS will significantly decrease the false-positive rate compared to traditional TPW without a significant increase in the false-negative rate.

Significant hepatic artery stenosis (HAS) after liver transplantation (LT) is thought to predispose patients to hepatic artery thrombosis, biliary complications and graft loss, which are associated with poor patient survival [1–3]. Operative intervention, re-transplantation or endovascular therapy may be a solution to HAS, and early detection is crucial to successful treatment [4–9].

Doppler ultrasonography (DUS) is the established method for the initial screening of vascular abnormalities after LT, even during the immediate postoperative period. The DUS diagnostic criteria for significant HAS after LT has withstood the test of time since Dodd III et al. [10] evaluated quantitative parameters derived from the main hepatic artery. Their criteria consist of a tardus parvus waveform [TPW, with resistive index (RI) < 0.5, systolic acceleration time (SAT) > 0.08 s] and peak systolic velocity (PSV) > 2 m/s. Subsequent research further extended to intrahepatic arteries [11–17].

Despite their widespread use and clinical utility, these criteria continue to present a diagnostic dilemma for clinicians. Since successfully detecting the PSV in the region of stenosis is very difficult to achieve, given its deep location and poor display on DUS, TPW is usually the preferred criterion for evaluating HAS. Nevertheless, a relatively high rate of false-positive diagnosis of HAS based on TPW has been reported as 11.2%–27.0% [10, 18], resulting in diverse management, depending on physicians or institutions, including immediate angiography or follow-up.

At our institution, additional liver function test results are used to further decide on the management strategy for patients with TPW (Fig. 1). Such a strategy reduces the false-positive cases but inevitably delays management for HAS patients with unapparent elevated liver function. Therefore, the purpose of this study is to determine clinical features and new Doppler cut-off values to provide strong evidence to diagnose HAS to help guide treatment with immediate angiography or follow-up.

**Fig. 1:**
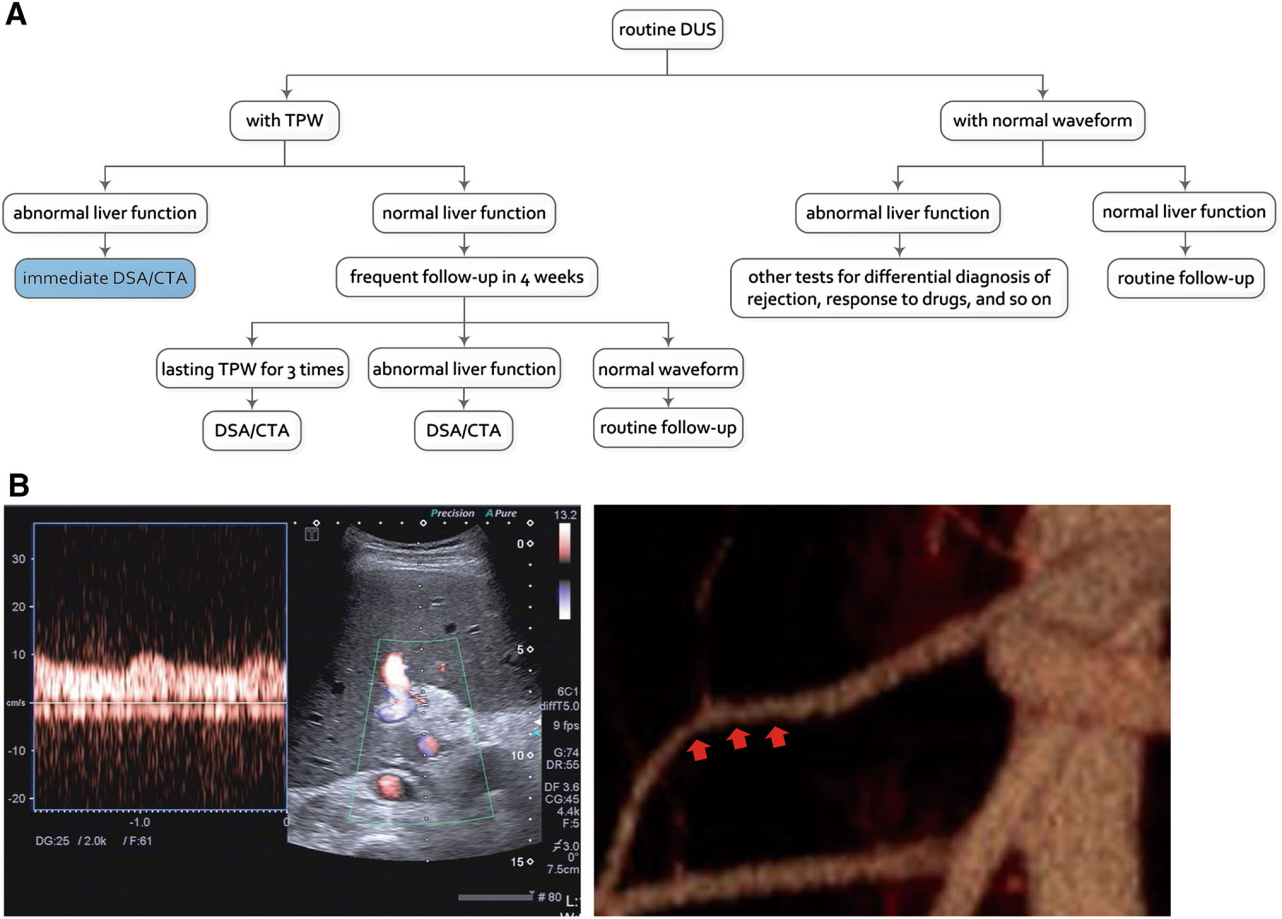
The current strategy at our institution to determine the treatment of a patient with tardus parvus waveform (TPW) on Doppler ultrasonography (DUS) after liver transplantation (LT) and an example. **A** The current strategy at our institution to determine the treatment of a patient with TPW on DUS. Additional liver function test results are added to help determine the need for immediate management (blue box). TPW is defined as resistive index (RI) < 0.5 and systolic acceleration time (SAT) > 0.08 s. The additional liver function is defined as any liver enzyme > 3-fold of the upper limit of normal level (ULNL) or 2-fold increased. **B** A case that met the current criteria to receive immediate treatment. Male, 39 years old, 2 months after LT due to liver cirrhosis associated with hepatitis B. TPW of the right hepatic artery (RHA) shown on DUS, with RI = 0.31 and SAT = 0.07 s (left). AST was 9-fold, and ALT was 14-fold of the ULNL on the same day. The patient met the current criteria to receive immediate treatment, and immediate CTA was performed the next day, which showed a HAS stenosis of 51.0% (right red arrow)

## Materials and methods

A total of 1654 liver transplants were performed in patients between March 2004 and March 2015. Patients were included in the study if they underwent DUS and a follow-up computed tomography angiography (CTA) or digital subtraction angiography (DSA) within a 4-week interval at least 1 month after the LT for a targeted evaluation of the hepatic arteries based on the present diagnostic procedure at our institution, as mentioned above (Fig. 1). If there were multiple examinations, the earliest pair was selected for each liver. A total of 190 transplanted livers in 189 patients fulfilled the inclusion criteria. Examination pairs were excluded for the following reasons: undetectable flow signal in both left and right hepatic arteries or quantitative parameters derived despite the documentation of arterial flow (*n* = 13); and unavailability of angiographic images in our picture archiving and communication system (PACS) (*n* = 5). The remaining 172 transplanted livers in 171 patients (total mean age 47 years ± 12, age range 7–71 years), including 146 men (mean age 47 years ± 11, age range 7–71 years) and 25 women (mean age 44 years ± 13, age range 16–64 years) (*P* = 0.334 for sex difference), constituted our study population. One patient underwent two LTs in an interval of 56 months. Out of these patients, 161 underwent deceased-donor LT, and the remaining patients underwent living-donor LT using modified right-lobe (*n* = 8) or left-lobe (*n* = 1) grafts. Indications for LT included hepatocellular carcinoma (*n* = 88), liver cirrhosis associated with hepatitis B or C virus (*n* = 32), fulminant hepatic failure (*n* = 39), Wilson’s disease (*n* = 2), alcoholic liver disease (*n* = 3), autoimmune liver disease (*n* = 2), polycystic liver disease (*n* = 1), congenital cirrhosis (*n* = 1), hepatoblastoma (*n* = 1), biliary stricture (*n* = 2), and liver metastases (*n* = 1).

The standard hepatic arterial reconstruction technique at our institution was end-to-end anastomosis using monofilament polypropylene sutures (Prolene, Ethicon) between the celiac trunk of the donor and the recipient common hepatic artery (CHA). Alternatively, a branch patch arterial anastomosis at the takeoff of the gastroduodenal artery from the CHA was performed. If the recipient hepatic artery was unsuitable for arterialization, an infra-renal aortic conduit derived from the donor iliac artery was anastomosed in an end-to-side fashion from the recipient aorta, and end-to-end anastomosis between the conduit and the celiac trunk of the donor was performed. In cases of hepatic artery anatomic variants involving the donor liver, complex hepatic arterial reconstructions were required that included the utilization of the superior mesenteric artery from the donor as the inflow vessel.

### DUS and analysis

All DUS examinations were performed using one of the US devices (Sequoia 512, Siemens, USA; Aplio 500, Toshiba, Japan) with a 3–5 MHz convex array transducer. To standardize how the spectral waveform and measurements were recorded, all DUS examinations were performed by one of three experienced radiologists (R.Z., J.R. and T.W., with 11, 10 and 4 years of experience in hepatic transplant imaging, respectively). Color and spectral Doppler sonograms of the peripheral right and left hepatic arteries were obtained. The standard Doppler parameters were adjusted to maximal gain to limit background noise, the lowest pulse-repetition frequency without aliasing artifacts, and a 2–5 mm Doppler sample gate for optimal signal detection from the common, left and right hepatic arteries of each liver. In arterial waveforms that were reproducible for at least three waveforms, the RI and SAT were calculated and documented.

The three radiologists recorded the RI and SAT of each hepatic artery and considered the presence of TPW with RI < 0.5 and SAT > 0.08 s in any hepatic artery of each liver [10]. The radiologists also recorded the minimal RI and maximum SAT of each liver.

### Reference findings and analysis

The liver function test results were also recorded for each patient within 4 weeks of DUS examination. The abnormal liver function suspicious for HAS was defined as any liver enzyme >3-fold of the upper limit of normal level (ULNL) or double increased. The mean interval between DUS and a follow-up CTA or DSA was 5.4 days ± 6.1 (range 0–29 days) without treatment.

Abdominal CTA was performed with one of the multi-detector CT (Light Speed QX/1, GE, USA; Aquilion One, Toshiba, Japan). The scanned area was from the subdiaphragmatic area to the lower hepatic rim. An aliquot of 0.8–2.0 mL/kg iodinated contrast medium (Ultravist 370 mg/mL; Bayer, Germany) was administered at a rate of 4–6 mL/s. Reconstruction of the hepatic artery imaging was performed via specific workstations (Advantage Windows 4.1, Sun Microsystems, USA; Aquilion One Displaying Monitor Workstation, Toshiba, Tokyo) for each patient. DSA (Multistar, Siemens, Germany) was performed after selective catheterization of the hepatic artery, and 150–250 mL non-ionic contrast medium (Omnipaque 300 mg/mL; Nycomed Amersham, Norway) was used. Hepatic arteriography was at 2 images/s.

A total of 155 CTA and 17 DSA were utilized as the reference. All angiograms were retrospectively reviewed by a radiologist (X.M., with 11 years of experience in hepatic transplant imaging) without knowledge of the DUS findings. HAS was defined as a short segment of narrowing and was determined to be clinically important if the region of stenosis was ≥50% in diameter. Angiographic measurements were made using calipers available on a PACS station on magnified axial or coronal images in the areas of stenosis and were compared with the normal-caliber artery identified more distally. The percentage of HAS was calculated as [(*D*_postHA_ − *D*_stenosisHA_)/*D*_postHA_] × 100% [18], where *D*_postHA_ is the diameter of the post to the stenosis segment of the hepatic artery, and *D*_stenosisHA_ is the diameter of the hepatic arterial stenosis. Accordingly, the 172 transplanted livers in 171 patients were assigned to HAS and non-HAS groups.

### Statistical analysis

Statistical analyses were performed using MedCalc (version 9.3.6.0, Ostend, Belgium) and SPSS (version 15.0, Chicago, USA). To avoid potential confounders in the classification of HAS and non-HAS groups, the independent *t* test and *χ* ^2^ test were used. With CTA or DSA as the reference, we analyzed the multilevel likelihood ratios (LRs) to explore new cut-off values across the whole spectrum of RI and SAT values. The LR for each category was calculated by dividing the percentage of patients with the target condition (HAS) in that category by the percentage without the condition in that category. LRs above 10 or below 0.1 are considered to provide strong evidence for including or excluding diagnoses, respectively [19, 20]. To determine treatment with immediate angiography or follow-up, groups were stratified into three categories according to the combination of traditional TPW, abnormal liver function, and new cut-off values defined as follows: category 1, subjects with traditional criteria for TPW (RI < 0.5 and SAT > 0.08 s); category 2, subjects with TPW and abnormal liver function (any liver enzyme > 3-fold of ULNL or double increased); category 3, subjects with TPW and abnormal liver function, or with new cut-off values. The sensitivity, specificity, positive, and negative predictive values (PPV and NPV, respectively) and accuracy of each category were evaluated and compared. All reported *P* values are two-sided, and *P* < 0.05 was considered statistically significant.

## Results

### Characteristics of patients

Of the 172 liver transplants, 67 (39.0%) had HAS at the reference with the mean percentage of stenosis of 75.4% (range 50%–100%). The remaining 105 (61.0%) were assigned to the non-HAS group. The major clinical parameters of the liver transplants included in the study are listed in Table 1.

**Table 1:** Characteristics of patients

Variables	HAS (*n* = 67)	Non-HAS (*n* = 105)	*P*
Age (years)	46 ± 10	47 ± 13	0.07
Male gender [*n* (%)]	59 (88.1)	88 (83.8)	0.51
Deceased-donor liver transplantation [*n* (%)]	65 (97.0)	98 (93.3)	0.33
TPW [*n* (%)]	42 (62.7)	7 (7.7)	< 0.01
Minimal RI	0.45 ± 0.13	0.62 ± 0.10	< 0.01
Maximum SAT (s)	0.11 ± 0.04	0.07 ± 0.02	< 0.01

### Analysis of optimal cut-off value

**Fig. 2:**
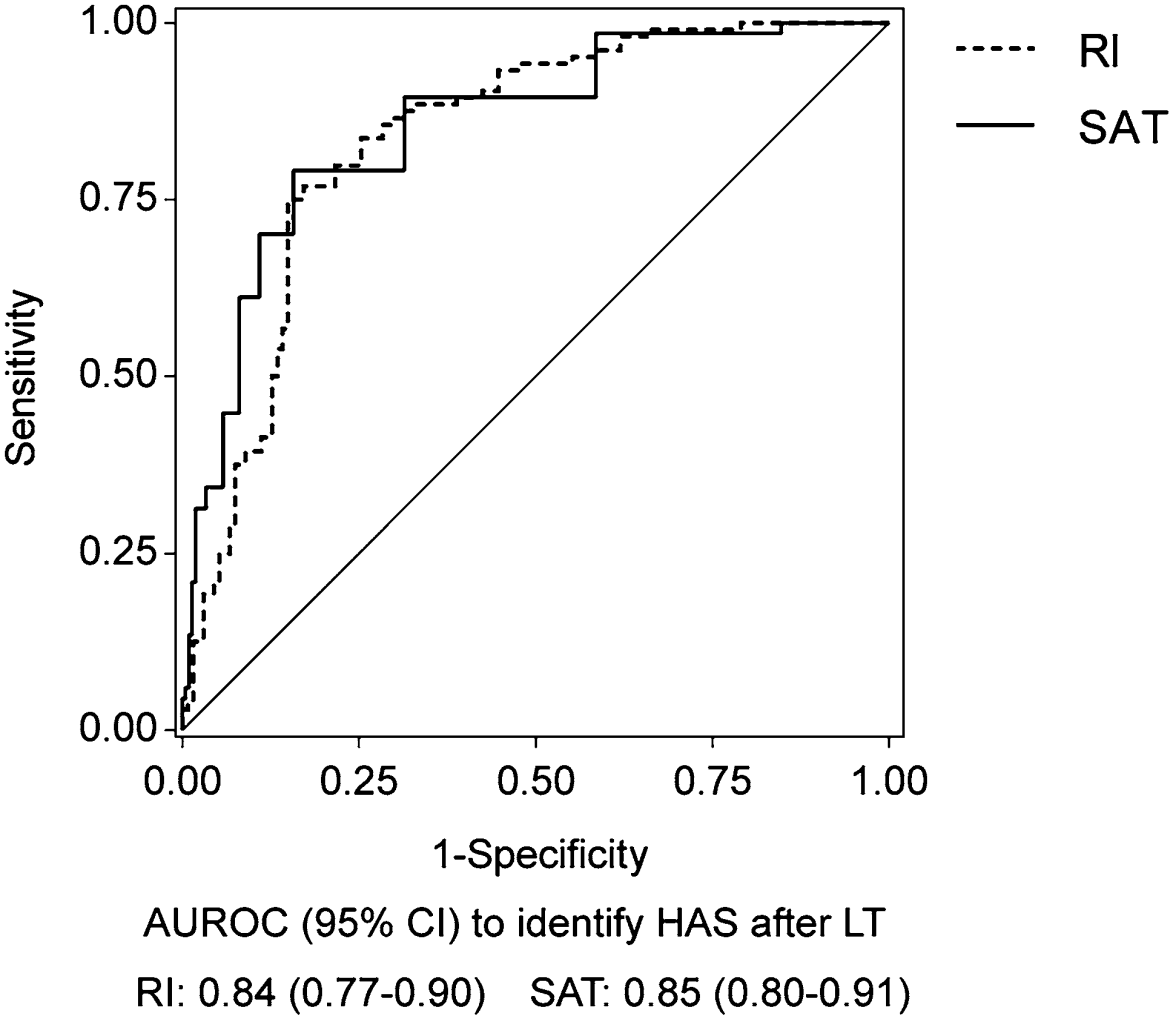
Diagnostic accuracy (AUROC) of RI and SAT to identify HAS after LT

The mean minimal RI was significantly lower in the HAS group (0.45 ± 0.13, range 0.23–0.77) than in the non-HAS group (0.62 ± 0.10, range 0.32–0.86, *P* < 0.001), and the mean maximum SAT was significantly longer in the HAS group (0.11 ± 0.04 s, range 0.05–0.23 s) than in the non-HAS group (0.07 ± 0.02 s, range 0.03–0.19 s, *P* < 0.001). The AUROC of RI and SAT for identifying HAS after LT was 0.84 (95% CI 0.77–0.90) and 0.85 (95% CI 0.80–0.91), respectively (Fig. 2).

Of the 172 liver transplants, 69 (40.1%) were diagnosed with TPW on DUS. The LR analysis revealed that the new cut-off values of RI < 0.4 (LR = 10.58) or SAT > 0.12 s (LR = 16.46) were adequate for including HAS (Table 2). Of the 172 liver transplants, 40 (23.3%) met the new cut-off values.

**Table 2:** Multilevel likelihood ratios (LRs) analysis for the prediction of hepatic arterial stenosis (HAS)

Minimum RI	LR (95% CIs)	Maximum SAT (s)	LR (95% CIs)
< 0.40*	10.58 (4.03–28.88)	≤ 0.06	0.19 (0.10–0.38)
≥ 0.40 and < 0.50	3.52 (1.79–7.65)	> 0.06 and ≤ 0.08	0.66 (0.43–1.16)
≥ 0.50 and < 0.70	0.83 (0.57–1.35)	> 0.08 and ≤ 0.12	3.70 (2.09–6.99)
≥ 0.70	0.24 (0.13–0.65)	> 0.12*	16.46 (4.16–67.92)

Likelihood ratios (LRs) above 10 and below 0.1 provide strong evidence to rule in or rule out diagnoses, respectively

*Resistance index (RI) or systolic acceleration time (SAT) value intervals that allow the prediction of hepatic arterial stenosis (HAS) with a sufficient degree of confidence

95% CIs, 95% confidence intervals

### Comparison with different categories

As the categories defined above [category 1, subjects with traditional criteria for TPW (RI < 0.5 and SAT > 0.08 s); category 2, subjects with TPW and abnormal liver function (any liver enzyme > 3-fold of ULNL or double increased); category 3, subjects with TPW and abnormal liver function, or with new cut-off values (RI < 0.4 or SAT > 0.12 s)], the diagnostic utility of these three categories was shown in Table 3. The specificities of categories 2 and 3 were significantly higher (92.4% vs. 81.9%, *P* = 0.038; 98.1% vs. 81.9%, *P* < 0.001, respectively), and the false-positive rates were significantly lower (7.6% vs. 18.1%, *P* = 0.038; 1.9% vs. 18.1%, *P* < 0.001, respectively) than those of category 1; meanwhile, the sensitivity of category 2 was significantly lower (41.8% vs. 74.6%, *P* < 0.001; 41.8% vs. 61.2%, *P* = 0.038, respectively) than that of categories 1 and 3.

**Table 3:** The diagnostic ability of different categories

	TP	TN	FP	FN	Sensitivity (%)	Specificity (%)	PPV (%)	NPV (%)	Accuracy (%)	False-positive rate (%)
Category 1	50	86	19	17	74.6	81.9	72.4	83.5	79.1	18.1
Category 2	28	97	8	39	41.8	92.4	77.8	71.3	72.7	7.6
Category 3	41	103	2	26	61.2	98.1	95.3	79.8	83.7	1.9

Category 1 is defined as subjects with traditional tardus parvus waveform (TPW). Category 2 is defined as subjects with TPW and abnormal liver function. Category 3 is defined as subjects with TPW and abnormal liver function, or with new cut-off values. TPW is defined as RI < 0.5 and SAT > 0.08 s of right or left hepatic artery. Abnormal liver function is defined as any liver enzyme > 3-fold of the upper limit of normal level (ULNL). The new cut-off values are defined as RI < 0.4 or SAT > 0.12 s of right or left hepatic artery

### Proposed strategies

**Fig. 3:**
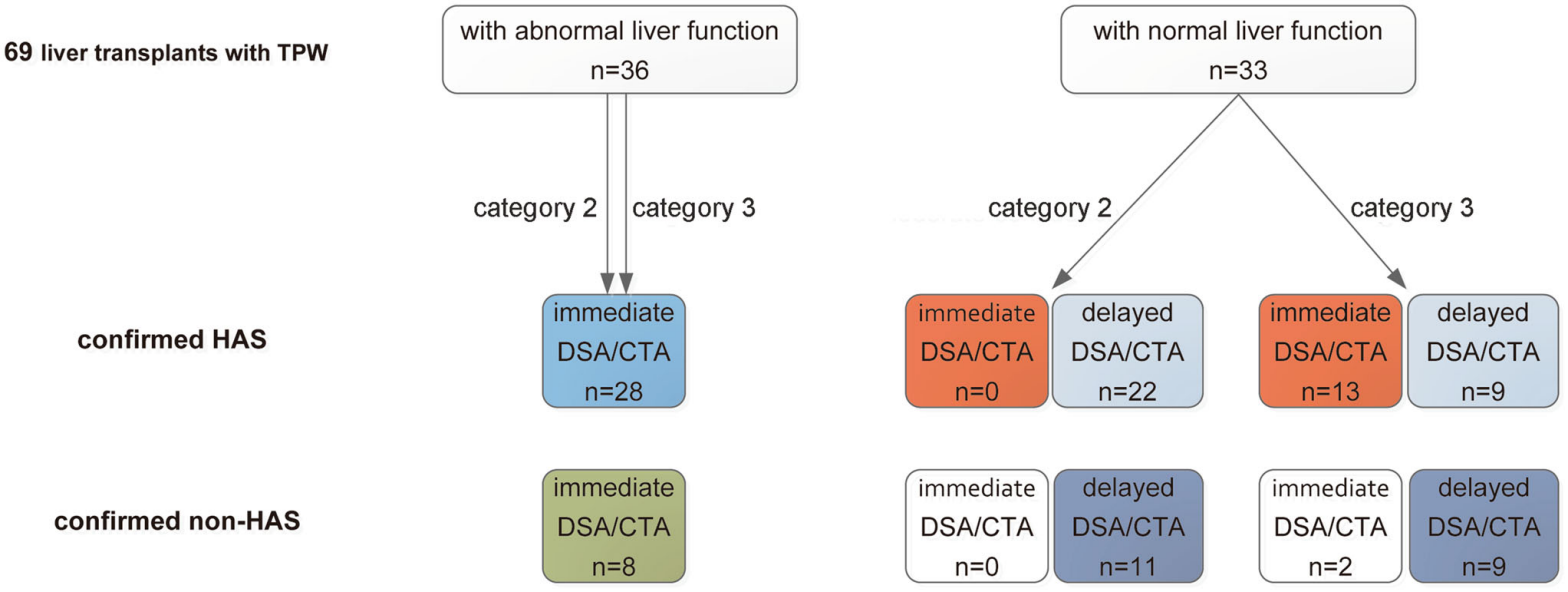
Flow chart with the diagnostic field of category 2 (combination of TPW and abnormal liver function) and category 3 (combination of TPW and abnormal liver function, or new cut-off values). When considering category 3, 13 more HAS patients (13/22, 59.1%) were correctly diagnosed with HAS (red boxes), decreasing the number of HAS patients who received delayed management from 22 to 9 (light blue boxes); while 2 more non-HAS patients (2/11, 18.2%) were misdiagnosed with HAS, increasing the non-HAS patients who received immediate management from 8 to 10 (white boxes)

**Fig. 4:**
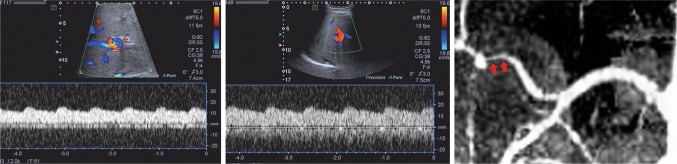
Case meeting category 3 and proven to be HAS on CTA. Male, 34 years old, 3 months after LT due to liver cirrhosis associated with hepatitis B. TPWs were shown on DUS, with RI = 0.29 and SAT = 0.11 s of the right HA (left), and RI = 0.30 and SAT = 0.11 s of the left HA (middle). Although both AST and ALT were normal on the same day, the patient insisted on further examination, and immediate CTA was performed on the same day, which showed HAS stenosis of 95.0% (right red arrow)

Although the combination of TPW and abnormal liver function (category 2) for depicting HAS has been adopted to decide on immediate CTA or DSA at our institute (Fig. 1), due to the high false-positive rate of TPW, 22 of 50 (44.0%) HAS patients in this study that met the category were misdiagnosed and were, therefore, unable to receive immediate management due to normal liver function (Figs. 3, 4). However, when considering category 3, 13 of these 22 HAS patients (59.1%) could be correctly diagnosed as HAS because they met the new cut-off values for DUS, which indicated that the number of HAS patients to receive immediate management would increase from 28 (28/50, 56.0%) to 41 (41/50, 82.0%) and delayed management would decrease from 22 (22/50, 44.0%) to 9 (9/50, 18.0%). In addition, category 3 would result in misdiagnosing HAS in 2 of the 11 non-HAS patients (18.2%), which indicated that the number of non-HAS patients receiving immediate management would slightly increase from 8 (42.1%) to 10 (52.6%).

## Discussion

We found that applying the traditional TPW criterion defined by Dodd III et al. [10] as a TPW with RI < 0.5 and SAT > 0.08 s indeed led to a high false-positive rate of 18.1%, which is identical to that in previous studies [10, 18]. The high false-positive rate indicates that a number of patients with TPW should not require treatment or further examinations. To avoid unnecessary management, the combination of TPW and additional abnormal liver function (category 2) for depicting HAS is now applied at our institution (Fig. 1). The false-positive rate indeed decreased to 7.6%. However, sensitivity also decreased from 74.6% to 41.8%, possibly because of a slow progression of these HAS cases with unapparent evaluated liver function within a few days, which also caused delayed management for these high-risk patients in our research.

To accurately diagnose HAS and to determine timely treatment or further examination for HAS patients, we analyzed multilevel LRs to establish new cut-off values for DUS to include or exclude HAS patients [19, 20], rather than the ROC analysis used in the previous studies [18, 21]. This statistical analysis has been successfully applied in several works to reliably indicate the disease stage without requiring invasive references [22]. The advantage of this approach is that unlike using sensitivity, specificity, and predictive values, it can be used to calculate the risk of abnormality without depending on the prevalence of abnormality for particular test results [19]. In this approach, LRs above 10 or below 0.1 are considered to provide strong evidence to include or exclude diagnoses, respectively [19, 20]. According to this approach, a RI < 0.4 or a SAT > 0.12 s strongly predicts patients with HAS (both LRs > 10), and thus, immediate management should be required, rather than waiting for elevated liver function.

Based on the results, we suggested new criteria (category 3, combination of TPW and abnormal liver function, or new cut-off values) for predicting HAS to improve clinical strategy. Compared with category 2 (combination of TPW and liver function), this category would correctly diagnose 13 more of the 50 HAS patients (from 56.0% to 82.0%), without significantly increasing the number of incorrect diagnoses of non-HAS patients (from 42.1% to 52.6%), which may be especially useful in HAS patients with normal liver function.

Studies on enhancing the utility of TPW for HAS have been reported, including an additional PSV [10, 18] or waveform grading system [22] as a solution. The initial criteria proposed by Dodd III et al. [10] also included a PSV threshold > 2 m/s besides TPW, and the combined parameters yielded 97% sensitivity and 64% specificity for marked arterial disease. However, Park et al. [18] suggested a contrary PSV threshold ≤ 48 cm/s, which improved the false-positive rate from 11.2% to 1.0% when combined with TPW. In addition, Choi et al. [22] proposed a TPW waveform grading system that achieved sensitivities of 78%–80% and specificities of 89%–91% when combined with RI < 0.5. However, these solutions appear to be suboptimal for clinical practice for the following reasons: (1) successfully detecting PSV in the region of stenosis is difficult to achieve because it is often difficult to identify the region of stenosis, usually at or near the anastomosis, due to its tiny arterial caliber and deep location or due to a poor sonic window and a discontinuous display of hepatic arterial flow. Even if a PSV is achieved, identifying whether it represents the real PSV in the region of stenosis or distal to stenosis is also difficult because the blood flow velocity increases initially in a critical stenosis and then rapidly decreases; (2) waveform pattern analysis is subjective; thus, attaining homologous results among different institutions worldwide may be difficult. In contrast, we proposed adding abnormal liver function and new cut-off values for DUS, which consisted of only RI and SAT (both of which are routinely obtained at most institutions). We believe that as routine parameters for DUS, RI, and SAT would be simpler and easier to adopt in practice than PSV and waveform pattern analysis to detect HAS in patients.

Our study also had several limitations. First, the design of retrospective studies is inherently limited in terms of the adjusted analysis that could be expected with a randomized controlled study. Instead, we compared age, sex, and liver-transplant type between the HAS and non-HAS groups as potential confounders, and no significant differences were found between these two groups (all *P* > 0.05). Second, the patients in the study were included over the course of a 10-year study period, resulting in the inclusion of the work of surgeons and sonographers with varying skill and experience and the results of various DUS machines and techniques. Relying on RI and SAT measurements by three different radiologists without assessing inter-observer variability is another limitation. In our study, all surgery and DUS examinations were performed using strict protocols that were already established for clinical purposes, including indication of types of arterial anastomosis and measurements of RI and SAT with accurate manual angle correction. Third, a verification bias is inherent in our study, wherein the patients with positive index test results selectively undergo the reference procedure, resulting in the exclusion of subjects with negative index test results and a higher diagnostic rate of HAS after LT of 39.0% (67/172) in our study, whereas the estimated incidence is reported to be 5%–9% in the literature [3, 23, 24]. This discrepancy is inevitable because it is difficult to routinely provide all patients with reference procedures. Fourth, most cases in our study used CTA (90.1%, 155/172) rather than DSA (9.9%, 17/172) as the reference. Although CTA and its reconstruction are reported to be well correlated with DSA in the diagnosis of HAS [25, 26], it is still a relatively imperfect reference compared with DSA. However, CTA is more commonly performed for the confirmative diagnosis of HAS at our institution because it is less invasive and correlates well with DSA in the diagnosis of HAS.

In summary, using either (1) RI < 0.4 or SAT > 0.12 s, or (2) traditional TPW (RI < 0.5 and SAT > 0.08 s) in the presence of abnormal liver functions as the DUS criteria for HAS will significantly decrease the false-positive rate compared to traditional TPW without a significant increase in the false-negative rate.
